# Exploring the Relationship Between *Clostridium thermocellum* JN4 and *Thermoanaerobacterium thermosaccharolyticum* GD17

**DOI:** 10.3389/fmicb.2019.02035

**Published:** 2019-09-10

**Authors:** Fangzhong Wang, Mingyu Wang, Qi Zhao, Kangle Niu, Shasha Liu, Didi He, Yan Liu, Shiping Xu, Xu Fang

**Affiliations:** ^1^State Key Laboratory of Microbial Technology, Shandong University, Qingdao, China; ^2^Center for Biosafety Research and Strategy, Tianjin University, Tianjin, China; ^3^College of Life Science, Qufu Normal University, Qufu, China; ^4^School of Environmental Science and Engineering, Shandong University, Qingdao, China

**Keywords:** *Clostridium thermocellum* JN4, *Thermoanaerobacterium thermosaccharolyticum* GD17, cellulose, biofuel, microbial consortia

## Abstract

Characterizing and engineering microbial communities for lignocellulosic biofuel production has received widespread attention. Previous research has established that *Clostridium thermocellum* JN4 and *Thermoanaerobacterium thermosaccharolyticum* GD17 coculture significantly improves overall cellulosic biofuel production efficiency. Here, we investigated this interaction and revealed the mechanism underlying the improved efficiency observed. In contrast to the previously reported mutualistic relationship, a harmful effect toward *C*. *thermocellum* JN4 was observed in these microbial consortia. Although *T. thermosaccharolyticum* GD17 relieves the carbon catabolite repression of *C*. *thermocellum* JN4 regarding obtaining more cellobiose or glucose released from lignocellulose, *T. thermosaccharolyticum* GD17 significantly hampers the growth of *C*. *thermocellum* JN4 in coculture. The increased formation of end products is due to the strong competitive metabolic advantage of *T. thermosaccharolyticum* GD17 over *C*. *thermocellum* JN4 in the conversion of glucose or cellobiose into final products. The possibility of controlling and rebalancing these microbial consortia to modulate cellulose degradation was achieved by adding *T. thermosaccharolyticum* GD17 stimulants into the system. As cellulolytic bacteria are usually at a metabolic disadvantage, these discoveries may apply to a large proportion of cellulosic biofuel-producing microbial consortia. These findings provide a reference for engineering efficient and modular microbial consortia for modulating cellulosic conversion.

## Introduction

A potentially imminent threat to mankind is our heavy reliability on fossil fuels for our energy, and the foreseeable depletion of these non-renewable resources (Turner, 1999; Shafiee and Topal, 2009). A solution to this problem is the development of technologies for the use of renewable energy sources, which include biomass based energy sources such as bioethanol (Rass-Hansen et al., 2007). Among all the available biomass reserves on earth, lignocellulose is the most abundant yet overwhelmingly underutilized due to its strong resistance against microbial and enzymatic degradation (Fang et al., 2010). It is therefore a priority to develop efficient and economically competitive technologies to manufacture lignocellulosic bioethanol, which has received widespread attention in the past few decades (Lynd et al., 1991).

The anaerobic thermophile *Clostridium thermocellum*, which forms a highly organized extracellular multi-enzyme complex, the cellulosome, can efficiently degrade cellulose (Demain et al., 2005; Gold and Martin, 2007; Balch et al., 2017; Singer et al., 2018). Furthermore, *C. thermocellum* integrates lignocellulose degradation and biofuel production by directly degrading cellulose to biofuels, eliminating the need for additional sugar-consuming, biofuel-forming microbes in the biofuel industry (Demain et al., 2005; Lynd et al., 2005; Olson and Lynd, 2012). Therefore, this bacterium is one of economically candidate microbes for lignocellulosic biorefinery applications. However, efficient biofuel production from monocultures of *C. thermocellum* has not yet been achieved, even after years of effort (Minty et al., 2013). Engineering a microbial consortium comprising two *C. thermocellum* strains or *C. thermocellum* with other non-cellulolytic bacteria is a promising strategy for enhancing overall efficiency during the production of biofuels such as ethanol, H_2_ and acetone-butanol-ethanol (Kato et al., 2004; Geng et al., 2010; He et al., 2011; Wen et al., 2017; Xiong et al., 2018). In particular, our previous investigation showed that naturally co-isolated *C. thermocellum* JN4 and *Thermoanaerobacterium thermosaccharolyticum* GD17 strains can form a synergistic microbial system in which the production of both cellulosic bioethanol and biohydrogen are doubled; these findings suggest that *C. thermocellum* and non-cellulolytic bacteria such as *T. thermosaccharolyticum* may form strong natural interactions that could benefit lignocellulosic bioethanol production (Liu et al., 2008; Pang et al., 2018).

Investigating and controlling interactions between *C. thermocellum* and its non-cellulolytic companion bacteria in coculture is a prerequisite for further improving cellulosic biofuel production in coculture. Because of the apparent increase in bioethanol and biohydrogen production in coculture *versus C. thermocellum* monoculture, this interaction was previously hypothesized to be of a mutualistic nature (Mori, 1990; Demain et al., 2005; Kato et al., 2005; Lu Y. C. et al., 2013), although solid evidence to support this hypothesis is scarce. Using the *C. thermocellum* JN4-*T. thermosaccharolyticum* GD17 synergistic cellulosic bioethanol production system as a model system, we herein provide evidence of a harmful effect toward *C. thermocellum* when co-existing with its non-cellulolytic companion. Our results show that this synergistic cellulosic bioethanol producing system can be controlled by fine-tuning the interaction between *C. thermocellum* and its companion bacterium. We believe that this interaction mode may provide a reference for designing and constructing intricate microbial consortia for efficient cellulose conversion.

## Materials and Methods

### Strains and Chemicals

*Clostridium thermocellum* JN4 (CGMCC 1.5210) and *T. thermosaccharolyticum* GD17 (CGMCC 1.5209) were isolated from cellulosic materials by our lab (Liu et al., 2008). They are now deposited in the China General Microbiological Culture Collection Center (CGMCC).

Corncob was kindly provided by Longlive Bio-Technology Co., Ltd. (Yucheng, Shandong, China). Ground corncob was prepared by grinding 14 g of shredded corncob in a ball mill (Model PULVERISETTE 5, FRITSCH GmbH, Idar-Oberstein, Germany) for 1 h (200 rpm, 5 min grinding interval). The production of cellulase solutions from *Penicillium* JUA10-1 followed a previously described method (Liu et al., 2010). Resazurin was purchased from Sigma-Aldrich Co. Ltd. (St. Louis, MO, United States). All other chemicals were purchased from Sinopharm Chemical Reagent Co., Ltd. (Shanghai, China).

### Bacterial Growth

*Clostridium thermocellum* JN4, *T. thermosaccharolyticum* GD17 and the reconstructed coculture of the two bacteria were grown using CTFUD media in anaerobic tubes/serum bottles in an incubator at 60°C without agitation. The composition of CTFUD media is: sodium citrate tribasic dihydrate 3.0 g/L, ammonium sulfate 1.3 g/L, potassium phosphate monbasic 1.5 g/L, calcium chloride dihydrate 0.13 g/L, L-cysteine-HCl 0.5 g/L, MOPS sodium salt 11.6 g/L, magnesium chloride hexahydrate 2.6 g/L, ferrous sulfate heptahydrate 0.001 g/L, cellobiose 5.0 g/L, yeast extract 4.5 g/L, resazurin 0.5 ml/L (Olson and Lynd, 2012). The concentration of carbon source in the media was 0.5%, expect for growth on Avicel + dextrin or Avicel + sucrose for which the concentration of each substrate was 0.5%. For inoculation, 10% (v/v) of seed culture was added to each tube/bottle.

### Analytical Methods

Analysis of glucose, cellobiose, lactate, acetate and ethanol was carried out using a Hitachi (Tokyo, Japan) High-Performance Liquid Chromatography (HPLC) system and an Aminex HPX-87H column (7.8 × 300 mm, 9 μm particle size) from Bio-Rad Laboratories Inc. (Hercules, CA, United States).

Analysis of residual cellulose in the cultures was carried out using either acid hydrolysis or enzymatic hydrolysis approaches with cellulose or filter papers, respectively, as the substrate. The dry cellulose pellet was prepared as follows: the pellet was subsequently washed three times with water to remove residual sugars and then dried at 105°C for 4 h in an electric oven. Acid hydrolysis of the dried cellulose residues followed methods described elsewhere (Zeng et al., 2007). For enzymatic hydrolysis, a 5-ml enzymatic hydrolysis system containing the dried pellet, 4.5 ml cellulase from *Penicillium* spp. (9.5 FPU/ml activities, 17.3 mg/ml protein content) buffered in citrate buffer (50 mM, pH 4.8, containing 1% Na_3_N) and 500 μl citrate buffer was prepared. The enzymatic hydrolysis system was incubated for 6 days at 45°C, and the supernatant was extracted for glucose content determination.

Analyses of glucose, cellobiose, lactate, acetate, ethanol and residual cellulose in cellulose-grown *C. thermocellum* JN4 and *C. thermocellum* JN4-*T. thermosaccharolyticum* GD17 cocultures were performed in 80-ml cultures; for inoculation, 10% of the total volume of cellulose-grown seed cultures was used. Three individual biological replicates were carried out for each experiment. Analyses of glucose, cellobiose, lactate, acetate and ethanol content in cellobiose or glucose-grown *C. thermocellum* JN4 and *T. thermosaccharolyticum* GD17 were performed in 50-ml cultures; for inoculation, 10% of the total volume of cellobiose or glucose-grown seed cultures was used. Three individual biological replicates were carried out for each experiment. Determination of biomass accumulation for *C. thermocellum* JN4 and *T. thermosaccharolyticum* GD17 on sucrose or dextrin was performed by periodically measuring OD_600_ in three individual replicates. To compare cellulose degradation rates of cocultures grown on Avicel, Avicel + dextrin and Avicel + sucrose, we assayed residual cellulose using the acid hydrolysis approach. Three individual biological replicates were carried out for each experiment.

### Relationship Between OD_600_ and Intracellular Protein Content

To determine the relationship between OD_600_ and the intracellular protein content using three individual replicates, *C. thermocellum* JN4 and *T. thermosaccharolyticum* GD17 were grown in media containing 0.5% glucose until an OD_600_ of 0.8–0.9 was reached. The cultures were then diluted with media to a final OD_600_ of 0.8000, 0.6000, 0.4000, and 0.2000. The cells were centrifuged at 12,000 rpm for 10 min to pellet the bacteria, after which total proteins were extracted using B-Per^®^ Bacterial Protein Extraction Reagent (Thermo Fisher Scientific Inc., Waltham, MA, United States). Determination of protein concentrations was carried out using a BCA protein assay kit (Thermo Fisher Scientific Inc., Waltham, MA, United States).

### Biomass and Bacterial Ratio Determination

To analyze biomass in *C. thermocellum* JN4 monocultures and *C. thermocellum* JN4-*T. thermosaccharolyticum* GD17 cocultures grown using cellulose, cellulose + sucrose, cellulose + dextrin, or ground corncob with three biological replicates, 10 ml culture was centrifuged for 10 min at 12,000 rpm to pellet both the substrate and bacteria. The pellet was subsequently washed twice with double distilled water, and total proteins were extracted from using B-Per^®^ Bacterial Protein Extraction Reagent (Thermo Fisher Scientific Inc., Waltham, MA, United States). Determination of protein concentrations was carried out using a BCA protein assay kit (Thermo Fisher Scientific Inc., Waltham, MA, United States).

To assess the ratio of *C. thermocellum* JN4 and *T. thermosaccharolyticum* GD17 in coculture grown on cellulose, cellulose + sucrose, cellulose + dextrin or ground corncob with three biological replicates, total DNA was extracted from cocultures until mid-log phase (24 h for cellulose or ground corncob, 12 h for cellulose + sucrose, cellulose + dextrin) using a SoilGen DNA kit (Beijing CoWin Bioscience Co., Ltd., Beijing, China). 16S rDNA was then sequenced using a MiSeq or HiSeq2500 PE250 system (Illumina Inc., San Diego, CA, United States). The species and abundance of sequenced 16S rDNA were determined.

Biomass formation by *C. thermocellum* JN4 in mono- and cocultures with *T. thermosaccharolyticum* GD17 was calculated from the relationship between the OD_600_ value and intracellular protein content, the assayed total protein content in cultures, and the cell ratio between the two bacteria in coculture.

### Real-Time PCR

To determine the expression level of genes involved in lignocellulose degradation in *C. thermocellum* JN4 monoculture and *C. thermocellum* JN4-*T. thermosaccharolyticum* GD17 coculture, total RNA was extracted from mid-log phase mono- and cocultures grown in media containing 0.5% cellulose, glucose or cellobiose using an E.Z.N.A. Bacterial RNA kit. cDNA synthesis was carried out using a PrimeScript^TM^ RT reagent kit with gDNA Eraser (Perfect Real Time) from Takara Bio Inc. (Shiga, Japan). qPCR was performed using a Roche LightCycler 96 system (Roche Applied Science, Mannheim, Germany) with FastStart Essential DNA Green Master (Roche Applied Science, Mannheim, Germany) as the dye. The primers used for real-time PCR are listed in Supplementary Table S1. The *recA* gene was used as the housekeeping gene (Stevenson and Weimer, 2005). The relative transcription levels of genes involved in lignocellulose degradation were calculated using the 2^–ΔΔCt^ method (Livak and Schmittgen, 2001).

### Statistics

The two-tailed Student *t*-test was carried out to evaluate significant differences between two sets of data; *p* < 0.05 was considered statistically significant. A minimum of three replicates was performed for each experiment.

## Results

### Enhanced End-Product Formation in *C*. *thermocellum* JN4-*T. thermosaccharolyticum* GD17 Coculture

A coculture of *C. thermocellum* JN4 and *T. thermosaccharolyticum* GD17 was reconstructed by co-inoculation of both strains on cellulose-containing media at a 1:1 cell ratio. The reconstructed community was stabilized after at least 5 passages. Using primers targeting specific DNA sequences for both *C. thermocellum* JN4 and *T. thermosaccharolyticum* GD17 (Supplementary Table S1), we were able to detect both strains in the reconstructed coculture (Supplementary Figure S1), confirming its composition.

**FIGURE 1:**
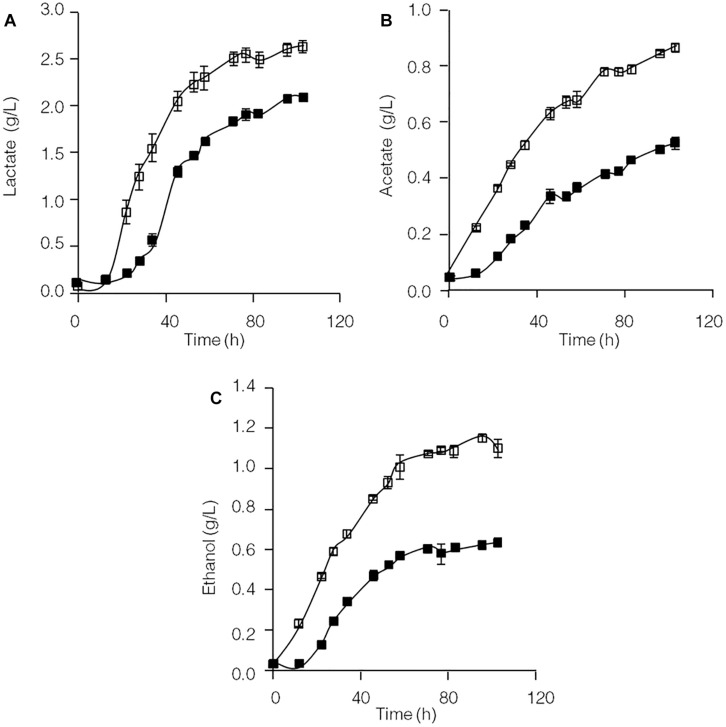
Production of lactate **(A)**, acetate **(B)**, and ethanol **(C)** in cellulose-grown *C. thermocellum* JN4 monoculture and coculture with *T. thermosaccharolyticum* GD17. Hollow square represents coculture, and solid square represents monoculture. Error bars represent standard errors calculated from three replicates.

Formation of the end products lactate, acetate and ethanol in the *C. thermocellum* JN4 monoculture and *C. thermocellum* JN4-*T. thermosaccharolyticum* GD17 coculture during growth on cellulose was evaluated. As shown in Figure 1, both the rate of formation and the final concentration of these end products of cellulose fermentation were clearly enhanced in the coculture. The initial rates of lactate, acetate and ethanol formation were increased by 63.9, 45.5, and 31.7%, respectively, and the final concentrations of lactate, acetate and ethanol were improved by 25.3, 73.1, and 84.7%, respectively.

### Derepression of Lignocellulose Degradation-Related Genes in Coculture Compared With Monoculture

**FIGURE 2:**
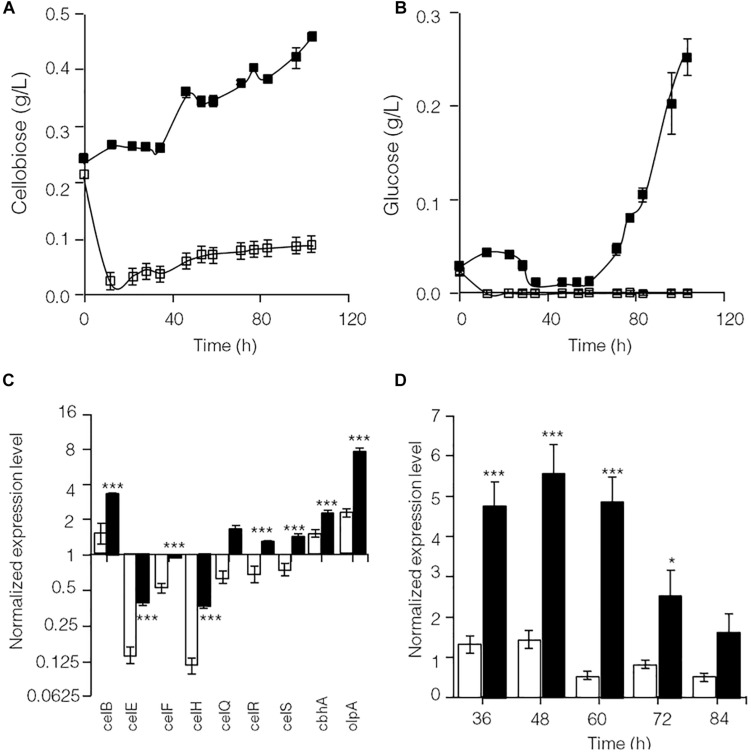
Cellulase inhibitor content and expression levels of key lignocellulose degradation-related genes in cellulose-grown *C. thermocellum* JN4 monoculture and coculture with *T. thermosaccharolyticum* GD17. **(A)** Cellobiose content. Hollow square represents coculture, and solid square represents monoculture. **(B)** Glucose content. Hollow square represents coculture, and solid square represents monoculture. **(C)** Gene expression in mid-log phase. Hollow represents monoculture, and solid represents coculture. **(D)** Kinetics of *celS* expression. Hollow represents monoculture, and solid represents coculture. Error bars represent standard errors calculated from three replicates for glucose and cellobiose contents and nine replicates for gene expression levels. Expression levels are normalized using *recA* expression as 1. ^∗^*p* < 0.05; ^∗∗∗^*p* < 0.001.

As shown in Supplementary Figure S2, glucose significantly repressed transcription of lignocellulose degradation-related genes in *C*. *thermocellum* JN4. Therefore, the levels of identified cellulase inhibitors, cellobiose (Zhang and Lynd, 2005b) and glucose, were examined in cellulose-grown *C. thermocellum* JN4 monoculture and *C. thermocellum* JN4-*T. thermosaccharolyticum* GD17 coculture (Figures 2A,B). After the residual cellobiose and glucose in the inoculum (at time zero) were rapidly consumed, much lower levels of both inhibitors were present in the coculture than in the monoculture. In particular, the level of glucose in the coculture was constantly zero. Consequently, the transcriptional levels of lignocellulose degradation-related genes in *C. thermocellum* JN4 were significantly higher in coculture than monoculture during mid-log phase growth on cellulose (24 and 48 h, respectively) (Figure 2C). Further detailed transcriptional analysis of *celS*, which encodes the most important cellulase component of the cellulosome in *C. thermocellum* JN4 showed constantly higher transcription in coculture during cellulose degradation (Figure 2D). All these results suggest that cellulase synthesis is significantly promoted in *C. thermocellum* JN4 when grown together with *T. thermosaccharolyticum* GD17 due to dampened levels of cellulase inhibitors in the coculture.

### Surprisingly Unchanged Cellulose Utilization in Coculture *versus* Monoculture

**FIGURE 3:**
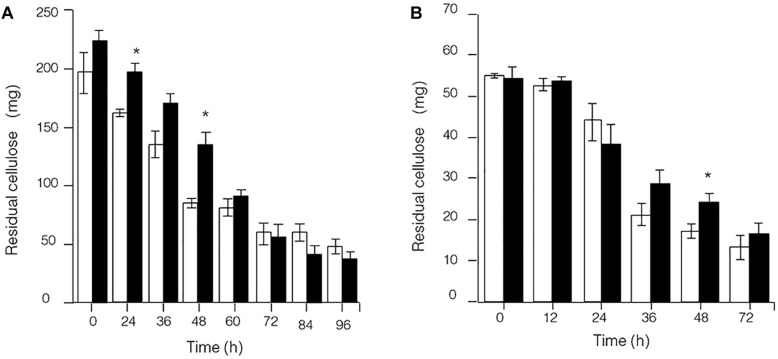
Presence of residual cellulose in cellulose-*C. thermocellum* JN4 monoculture and coculture with *T. thermosaccharolyticum* GD17. Hollow represents monoculture, and solid represents coculture. **(A)** Residual cellulose determined using acid hydrolysis. **(B)** Residual cellulose determined using enzymatic hydrolysis. Error bars represent standard errors calculated from three replicates. ^∗^*p* < 0.05.

Because the synthesis of lignocellulose degradation-related genes in *C. thermocellum* JN4 was upregulated when grown together with *T. thermosaccharolyticum* GD17, we originally expected that if true mutualism exists between *C. thermocellum* JN4 and *T. thermosaccharolyticum* GD17, substrate utilization by *C. thermocellum* JN4 would also be promoted. This reasoning is because *C. thermocellum* JN4 supplies *T. thermosaccharolyticum* GD17 with the growth substrates glucose and cellobiose and consumes these substances that inhibit cellulose degradation in *C. thermocellum* JN4 thus benefiting *C. thermocellum* JN4 by promoting substrate consumption. Our analysis of cellulose degradation in mono- and coculture, however, suggested a different scenario. Evaluation of residual cellulose in cellulose-grown *C. thermocellum* JN4 monoculture and *C. thermocellum* JN4-*T. thermosaccharolyticum* GD17 coculture was carried out either by (1) degrading residual cellulose in the culture with acid or (2) degrading residual cellulose in the culture with *Penicillium* cellulases, followed by analysis of evolved glucose. Surprisingly, no significant difference in residual cellulose content and degradation rate between monoculture and coculture was observed using either method (Figure 3). Furthermore, the content of residual cellulose was high in the coculture at 24 or 48 h when using the acid hydrolysis method (Figure 3A). These findings were in contrast to our original expectation and the mutualism model.

### *T. thermosaccharolyticum* GD17 Significantly Hampers the Growth of *C. thermocellum* JN4 on Cellulose and Corncob

**FIGURE 4:**
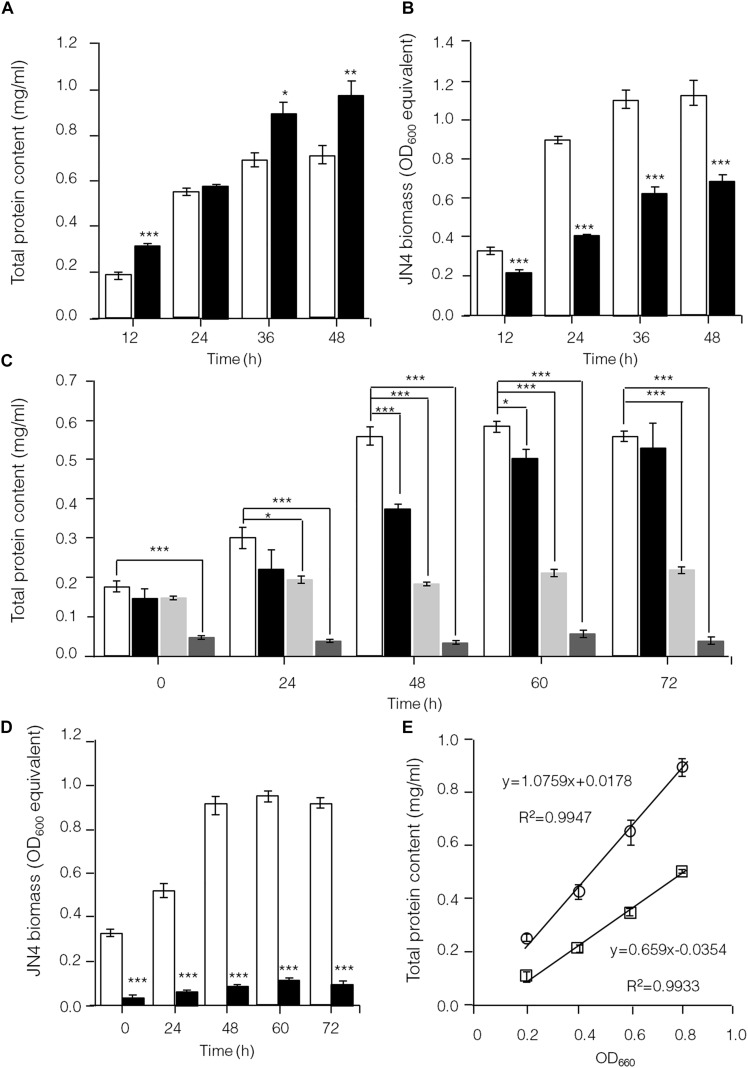
Hampered *C. thermocellum* JN4 growth in coculture with *T. thermosaccharolyticum* GD17. **(A)** Growth curve (with total protein content) of *C. thermocellum* JN4 monoculture and coculture with *T. thermosaccharolyticum* GD17 on cellulose. Hollow represents *C. thermocellum* JN4 monoculture, and solid black represents coculture. **(B)** Biomass of *C. thermocellum* JN4 in monoculture and cocultures grown on cellulose. Hollow represents monoculture, and solid black represents coculture. **(C)** Growth curve (with total protein content) of *C. thermocellum* JN4, *T. thermosaccharolyticum* GD17 and their coculture on ground corncob. Hollow represents *C. thermocellum* JN4 monoculture, and solid black represents coculture. Light gray represents *T. thermosaccharolyticum* GD17 monoculture with cellobiose-grown culture as inoculum. Dark gray represents *T. thermosaccharolyticum* GD17 monoculture with corncorb-grown culture as inoculum. **(D)** Biomass of *C. thermocellum* JN4 in monoculture and coculture grown on ground corncob. Hollow represents monoculture, and solid black represents coculture. **(E)** Relationship of OD_600_ and total protein content. Open square: *C. thermocellum* JN4, Open circle: *T. thermosaccharolyticum* GD17. Error bars represent standard errors calculated from three replicates. ^∗^*p* < 0.05; ^∗∗^*p* < 0.01; ^∗∗∗^*p* < 0.001.

To further identify interactions between *C. thermocellum* JN4 and *T. thermosaccharolyticum* GD17 in coculture, we performed a biomass analysis of these microbes. Considering that the shape and size of *C. thermocellum* JN4 and *T. thermosaccharolyticum* GD17 are nearly identical (almost indistinguishable by microscopy), we assumed that the same number of cells for each strain would lead to the same optical density at 600 nm. We then quantified the relationships between OD_600_ value and intracellular protein concentration for each bacterium, which suggested a very good linear relationship (Figure 4E) and also indicated that the intracellular protein content is as a good measure of biomass. Based on this relationship, the biomass of *C. thermocellum* JN4 in coculture can be assayed by determining the total intracellular protein content in coculture if the cell ratio between *C. thermocellum* JN4 and *T. thermosaccharolyticum* GD17 is known.

*Clostridium thermocellum* JN4, *T. thermosaccharolyticum* GD17 and their coculture were grown on cellulose and ground corncob, resembling natural substrates. Robust growth of *C. thermocellum* JN4 on both substrates was observed (Figures 4A,C). Conversely, when using cellobiose or corncob-grown *T. thermosaccharolyticum* GD17 as seed cultures, *T. thermosaccharolyticum* GD17 did not grow on cellulose, with only weak growth on corncob (Figure 4C). This result is in agreement with a previous report that *T. thermosaccharolyticum* GD17 can utilize hemicellulose, which is present in corncob, but not cellulose (Chimtong et al., 2011). Therefore, *C. thermocellum* JN4 appears to provide glucose and cellobiose, which is derived from cellulosic degradation, to *T. thermosaccharolyticum* GD17, and *T. thermosaccharolyticum* GD17 may also benefit *C. thermocellum* JN4 by digesting hemicellulose to xylose.

The analysis of intracellular protein contents of *C. thermocellum* JN4 monoculture and coculture with *T. thermosaccharolyticum* GD17 showed similar patterns of biomass accumulation (Figures 4A,C). We further identified the ratio of *C. thermocellum* JN4 to *T. thermosaccharolyticum* GD17 cells in coculture grown on both cellulose and ground corncob by quantifying 16S rDNA for each microbe in genomic DNA extracted from the coculture using high-throughput sequencing. In cellulose-grown cocultures, 56.9 ± 3.6% (mean ± SEM, *n* = 3) of the cells are *C. thermocellum* JN4, and 42.7 ± 3.7% (mean ± SEM, *n* = 3) of the cells are *T. thermosaccharolyticum* GD17. In corncob-grown cocultures, 21.1 ± 7.5% (mean ± SEM, *n* = 3) of the cells are *C. thermocellum* JN4 and 78.9 ± 7.5% (mean ± SEM, *n* = 3) of the cells are *T. thermosaccharolyticum* GD17. Based on these results, we further determined the biomass present in coculture grown on either cellulose or ground corncob, and the results showed strong growth repression of *C. thermocellum* JN4 in both cases (Figures 4B,D).

### Competitive Metabolic Advantage of *T. thermosaccharolyticum* GD17 Over *C. thermocellum* JN4

**FIGURE 5:**
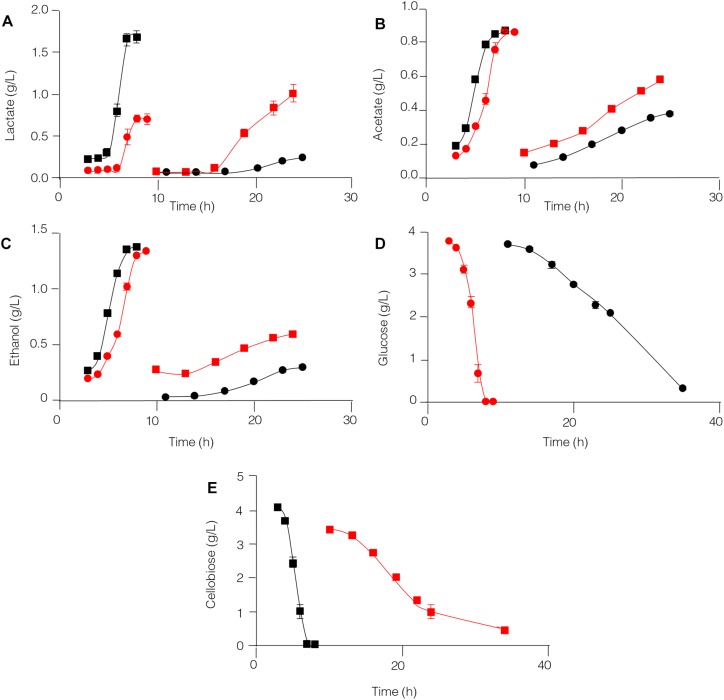
Glucose and cellobiose metabolism by *C. thermocellum* JN4 and *T. thermosaccharolyticum* GD17. **(A)** Lactate production. **(B)** Acetate production. **(C)** Ethanol production. **(D)** Glucose consumption. **(E)** Cellobiose consumption. Black circle represents *C. thermocellum* JN4 was grown on media containing glucose as the carbon source; red square represents *C. thermocellum* JN4 was grown on media containing cellobiose as the carbon source; red circle represents *T. thermosaccharolyticum* GD17 was grown on media containing glucose as the carbon source; black square represents *T. thermosaccharolyticum* GD17 was grown on media containing cellobiose as the carbon source. Error bars represent standard errors calculated from three replicates.

The capability of *C. thermocellum* JN4 and *T. thermosaccharolyticum* GD17 to degrade glucose and cellobiose and to produce lactate, acetate, and ethanol was compared (Figure 5). *T. thermosaccharolyticum* GD17 clearly has a competitive advantage over *C. thermocellum* JN4 on glucose and cellobiose, degrading these substrates at rates 8.27- and 6.17-fold, respectively, higher than those of *C. thermocellum* JN4. The rate of end-product formation was higher in *T. thermosaccharolyticum* GD17 than in *C. thermocellum* JN4 for lactate (15.63-fold on glucose and 5.57-fold on cellobiose), acetate (8.85-fold on glucose and 6.97-fold on cellobiose), and ethanol (11.92-fold on glucose and 10.60-fold on cellobiose). For each carbon atom in glucose, *C. thermocellum* JN4 transfers 0.53 ± 0.01 atoms (mean ± SEM, *n* = 3) to lactate, acetate and ethanol, lower than the 0.77 ± 0.07 atoms (mean ± SEM, *n* = 3, *p* = 0.026) of *T. thermosaccharolyticum* GD17. For each carbon atom in cellobiose, *C. thermocellum* JN4 transfers 0.89 ± 0.03 atoms (mean ± SEM, *n* = 3) to lactate, acetate and ethanol, lower than the 1.00 ± 0.03 atoms (mean ± SEM, *n* = 3, *p* = 0.026) of *T. thermosaccharolyticum* GD17. These results suggest that *T. thermosaccharolyticum* GD17 has more robust glucose and cellobiose metabolism than *C. thermocellum* JN4 and that it is more efficient in the conversion of glucose or cellobiose to the end-products lactate, acetate and ethanol. We can therefore conclude that *T. thermosaccharolyticum* GD17 has a clear and strong competitive metabolic advantage over *C. thermocellum* JN4 and that the better productive efficiency of *T. thermosaccharolyticum* GD17 is responsible for the improved alcohol and acid formation in coculture. This result is in contrast to the proposed mutualism between cellulolytic and non-cellulolytic bacteria.

### A Harmful Effect Toward *C. thermocellum* JN4 Was Observed When Co-existing With *T. thermosaccharolyticum* GD17

**FIGURE 6:**
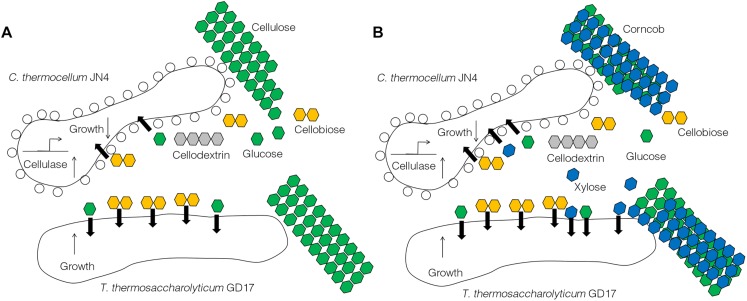
A tentative model of the interactions between *C*. *thermocellum* JN4 and *T*. *thermosaccharolyticum* GD17. **(A)** *C. thermocellum*/ *T. thermosaccharolyticum* coculture grown on cellulose. **(B)** *C. thermocellum*/*T. thermosaccharolyticum* coculture grown on ground corncob.

The results obtained from this work suggest *C. thermocellum* JN4 suffers from growth inhibition when co-existing with *T. thermosaccharolyticum* GD17 in the presence of cellulose or corncob, which is in stark contrast to a generally believed mutualism in which the two organisms benefit each other. Repression of *C. thermocellum* JN4 is the result of competition on nutritional substrates such as glucose and cellobiose and the failure of *C. thermocellum* JN4 to obtain sufficient nutrients in the presence of *T. thermosaccharolyticum* GD17, which consumes them at a rate 6–8-fold higher than *C. thermocellum* JN4. Although the presence of *T. thermosaccharolyticum* GD17 leads to repression relief of *C. thermocellum* JN4 regarding cellulase-coding genes, this derepression cannot compensate for the lack of nutrients and subsequently the decrease in biomass. A tentative model of the interactions between *C. thermocellum* JN4 and *T. thermosaccharolyticum* GD17 can be generated according to these observations (Figure 6).

### Re-balancing the Synthetic Microbial Consortium Comprising *C. thermocellum* JN4 and *T. thermosaccharolyticum* GD17 for Modulating Cellulosic Conversion

**FIGURE 7:**
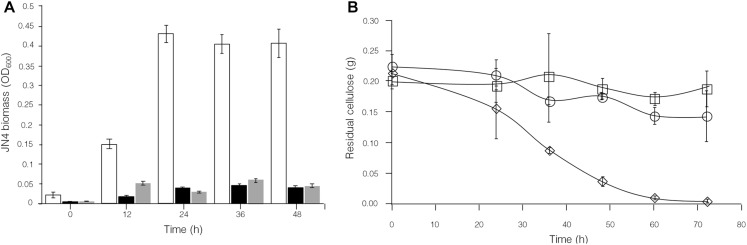
Comparison of *C. thermocellum* JN4 biomass accumulation and consumption of cellulose in *C. thermocellum* JN4/*T. thermosaccharolyticum* GD17 coculture grown on Avicel, Avicel + sucrose and Avicel + dextri. **(A)** *C. thermocellum* JN4 biomass accumulation. Hollow represents Avicel, black represents Avicel + sucrose and gray represents Avicel + dextri. **(B)** Residual cellulose content. Diamond represents Avicel, round represents Avicel + sucrose and square represents Avicel + dextrin. Biomass is represented in OD_600_ equivalents.

To control the interaction between *C. thermocellum* JN4 and *T. thermosaccharolyticum* GD17, two potential stimulants for *T. thermosaccharolyticum* GD17 were selected: dextrin and sucrose. Growth studies showed much stronger growth of *T. thermosaccharolyticum* GD17 compared to *C. thermocellum* JN4 on both substances (Supplementary Figure S3). Supplementation of either dextrin or sucrose to Avicel-grown cocultures of *C. thermocellum* JN4-*T. thermosaccharolyticum* GD17 was carried out, and growth analysis using high-throughput 16S rDNA sequencing clearly showed a dramatic decrease in the content of *C. thermocellum* JN4 in coculture (Figure 7A, from 48.4 ± 0.5% to 8.9 ± 0.2% when dextrin was supplemented and from 48.4 ± 0.5% to 5.2 ± 5.2% when sucrose was supplemented, mean ± SEM, *n* = 3). These results are in agreement with the stimulatory effects of these two substances on the growth of *T. thermosaccharolyticum* GD17 compared to *C. thermocellum* JN4. Consequently, the re-balanced cocultures largely stopped degrading cellulose (Figure 7B). These results confirm our finding that *T. thermosaccharolyticum* GD17 inhibits, rather than benefits, *C. thermocellum* JN4 growth in coculture. They also demonstrate the possibility of modulating cellulose degradation and subsequently cellulosic biofuel formation by controlling the interaction of these two bacteria and of re-balancing the microbial community structure in coculture.

## Discussion

Results obtained in this and previous work clearly suggests that the use of a coculture of *C*. *thermocellum* and a non-cellulolytic companion is an improved strategy on cellulosic bioethanol production over the application of *C*. *thermocellum* monocultures (Liu et al., 2008; Chang and Yao, 2011; He et al., 2011). In contrast to a previously assumed mutualistic model, we observed *T. thermosaccharolyticum* GD17 hindered *C. thermocellum* JN4 biomass accumulation when co-cultured with *T. thermosaccharolyticum* GD17 (Figures 4B,D), as assessed using a biomass determination technique. The total content of extracted protein from *C. thermocellum* JN4 or *T. thermosaccharolyticum* GD17 culture was linearly correlated with the OD_600_ (Figure 4E), which has also been applied and discussed previously for anaerobic microbes (Jensen et al., 2008; Holwerda et al., 2013). Quantitative 16S rDNA sequencing to evaluate the *C. thermocellum* JN4 and *T. thermosaccharolyticum* GD17 ratio has been used for analyzing microbial community composition and identifying new bacteria (Woo et al., 2008; Krober et al., 2009; Siddiqui et al., 2011).

It is well-known that glucose is an inhibitor on cellulase synthesis in many cellulosic degrading microorganisms, but this effect is different in *Clostridium* species. For example, glucose increased the cellulolytic enzyme production in *C*. *cellulolyticum* (Xu et al., 2013). On the contrary, in *C*. *cellulovorans*, glucose suppressed the expression of cellulase genes at the transcriptional level (Han et al., 2003) as same as that observed in *C*. *thermocellum* JN4 (Supplementary Figure S2). Therefore, the effect of glucose on cellulase synthesis should be carefully checked in *Clostridium*.

It has been thoroughly established that *C. thermocellum* prefers the transport of cellodextrin instead of cellobiose or glucose for metabolism (Zhang and Lynd, 2005a; Nataf et al., 2009). In addition to saving energy for carbohydrate transport, this phenomenon may also suggest the evolutionary adaptation of *C. thermocellum* to avoid metabolizing glucose and cellobiose directly, which can be viewed as a defense mechanism against non-cellulolytic bacterium. However, this defense mechanism can only partially recover the impaired growth of *C. thermocellum* because the rapid consumption of glucose and cellobiose by non-cellulolytic bacteria will thermodynamically promote more degradation of cellodextrin to glucose and cellobiose, therefore still effectively reducing the level of nutrients that *C*. *thermocellum* absorbs. As mentioned above, the concentration of cellodextrin fluctuated over time; therefore the type of interaction in this microbial consortium is far from straightforward. For example, when the amount of cellodextrin was elevated, it was indicated that *C*. *thermocellum* JN4 could absorb more amount of preferred carbon source, and then we speculated that there is an amensal relationship between *C. thermocellum* JN4 and *T. thermosaccharolyticum* GD17. When cellodextrin was almost exhausted, *C*. *thermocellum* JN4 lost the advantage over absorbing carbon source, leading to seriously damaged growth. Therefore, it was possible that there is a parasitism relationship between *C. thermocellum* JN4 and *T. thermosaccharolyticum* GD17. Anyway, a harmful effect toward *C*. *thermocellum* JN4 was observed when co-cultured with non-cellulolytic bacteria.

Additional analyses could find the contradictory on the previously hypothetical mutualism between *C. thermocellum* JN4 and *T. thermosaccharolyticum* GD17. In a hypothetical mutualistic relationship, *T. thermosaccharolyticum* GD17 can remove glucose and cellobiose for metabolism while benefiting *C. thermocellum* JN4 by lifting carbon catabolite repression induced by glucose and cellobiose yet leaving sufficient glucose and cellobiose for *C. thermocellum* JN4 to use; as the amount of substrate would be greater than when *C. thermocellum* JN4 is grown on cellulose in monoculture, it would grow better. However, leaving more glucose and cellobiose would defeat the purpose of “lifting cellulase synthesis inhibition” because more inhibition would occur in coculture than in monoculture. Thus, a paradox exists in a mutualistic relationship.

The observed relationship between *C. thermocellum* JN4 and *T. thermosaccharolyticum* GD17 led us to address previously observed but unexplained phenomena regarding the following: *C. thermocellum* is difficult to separate from accompanying non-cellulolytic bacteria (Freier et al., 1988; Yong-Eok et al., 1993; Erbeznik et al., 1997; Liu et al., 2008); the production of end products is improved in coculture of *C. thermocellum* and non-cellulolytic bacteria (Ng and Zeikus, 1982; Liu et al., 2008; Geng et al., 2010; He et al., 2011; Nakayama et al., 2011; Li and Liu, 2012; Lu Y. C. et al., 2013); *C. thermocellum* commonly occurs in the presence of cellulose in anaerobic microbial consortia (Luo et al., 2011; Smith et al., 2014). Two key principles should be highlighted during establishment of the relationship: (1) repression of cellulase synthesis by products of cellulose degradation; (2) competition at the thermodynamic level for the formation and consumption of products from cellulose degradation by different bacteria. We believe this relationship applies to a large proportion of cellulosic bioethanol-producing microbial systems because cellulolytic bacteria naturally have a competitive disadvantage regarding “easy” substrates such as glucose.

Based on these discoveries, to achieve maximal results, microbial consortia for modulating cellulosic production need to be carefully balanced between cellulose degradation and biofuel formation. More specifically, the four microbial processes, cellulose degradation by cellulolytic bacteria, repression of cellulolytic bacteria, depression of cellulase formation, and enhancement of product formation by non-cellulolytic bacteria, need to be carefully balanced for maximizing rates and efficiency in cellulosic biofuel formation.

Previously published works have reported approaches for balancing such microbial communities, primarily via bioaugmentation of additional microbes in natural or designer microbial communities (Lu F. et al., 2013; Peng et al., 2014; Martin-Ryals et al., 2015). In this work, we demonstrate an additional approach for controlling interactions between *C. thermocellum* JN4 and *T. thermosaccharolyticum* GD17 based the discovery: adding external stimulants for the microbes. We describe a successful attempt to stimulate the growth and alter the proportion of *T. thermosaccharolyticum* GD17 and subsequently changed cellulose degradation. Pending the discovery of more stimulants and inhibitors for both *C*. *thermocellum* and non-cellulolytic bacteria, we believe that structural optimization of the microbial system and subsequent optimization of cellulosic biofuel production may be achieved.

## Conclusion

These investigations reveal a new relationship between *C. thermocellum* and a non-cellulolytic companion, in contrast to the previously hypothesized mutualistic relationship, as well as the mechanism underlying improved cellulosic bioethanol formation, namely, that the non-cellulolytic bacterium outperforms *C. thermocellum* in sugar-to-ethanol conversion. We also proposed and verified an approach of stimulant addition for each bacterium in the microbial consortium to re-balance the microbial community structure. These discoveries serve as a basis for designing and optimizing microbial consortia between cellulolytic and non-cellulolytic bacteria to modulate cellulose conversion.

## Data Availability

All datasets generated for this study are included in the manuscript and/or the Supplementary Files.

## Author Contributions

XF, MW, and FW conceived the project and designed the experiments. XF, MW, and FW analyzed the data, and wrote and revised the manuscript. QZ, KN, SL, DH, and YL performed the experiments. All authors discussed the manuscript and agreed to publish.

## Conflict of Interest Statement

The authors declare that the research was conducted in the absence of any commercial or financial relationships that could be construed as a potential conflict of interest.

## Supplementary Material

FIGURE S1 Amplification of sequences specific to *C. thermocellum* JN4 and *T. thermosaccharolyticum* GD17. G: *T. thermocellum*; J: *C. thermocellum* JN4; C: Coculture of *T. thermosaccharolyticum* GD17 and *C. thermocellum* JN4. GD-1 and GD-2 are sequences specific to *T. thermosaccharolyticum* GD17. JN-1 and JN-2 are sequences specific to *C. thermocellum* JN4.

FIGURE S2 Comparison of expression levels of lignocellulose degradation related genes in *C. thermocellum* JN4 during growth on cellulose, cellobiose and glucose. Black represents cellulose, gray represents cellobiose and hollow represents glucose. Error bars are representation of standard errors calculated from nine replicates. Expression levels are normalized using *recA* expression as 1.

FIGURE S3 Growth of *C. thermocellum* JN4 and *T. thermosaccharolyticum* GD17 on dextrin and sucrose. **(A)** Growth on dextrin. **(B)** Growth on sucrose. Closed circle: *T. thermosaccharolyticum* GD17; closed square: *C. thermocellum* JN4. Error bars are representation of standard errors calculated from three replicates.

TABLE S1 Primers used in this study.
