# Supplementary figures and images for: Exploring the Relationship Between Clostridium thermocellum JN4 and Thermoanaerobacterium thermosaccharolyticum GD17
Source: Front Microbiol. 2019 Sep 10;10:2035. doi: 10.3389/fmicb.2019.02035 (PMC6746925; doi:10.3389/fmicb.2019.02035)

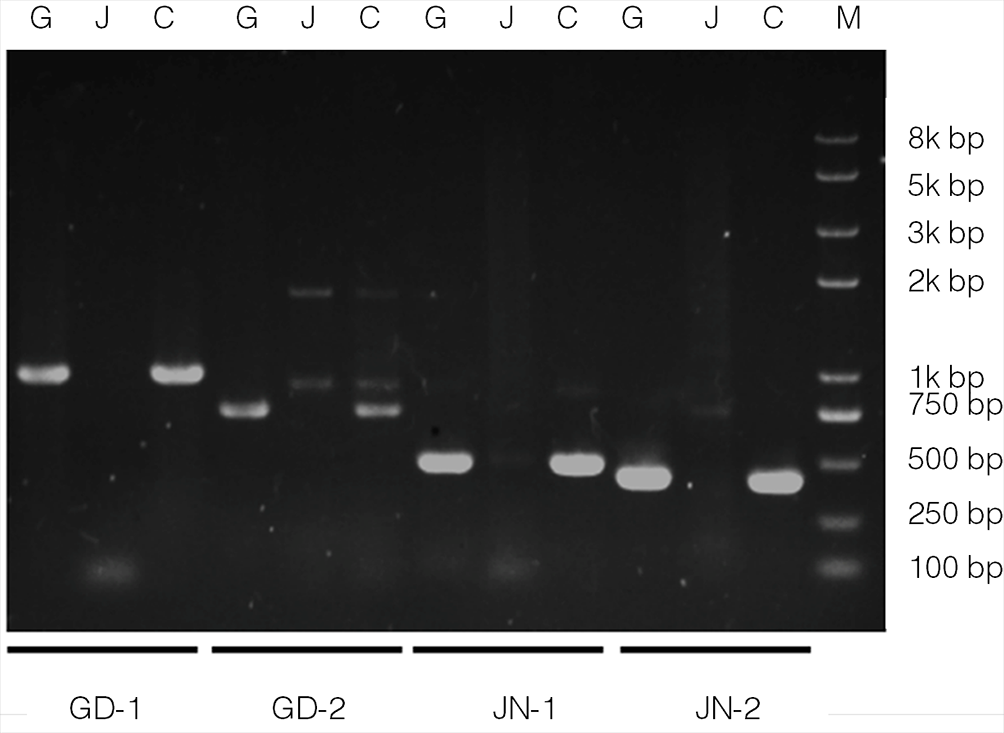

Supplement: FIGURE S1 — Amplification of sequences specific to C. thermocellum JN4 and T. thermosaccharolyticum GD17. G: T. thermocellum; J: C. thermocellum JN4; C: Coculture of T. thermosaccharolyticum GD17 and C. thermocellum JN4. GD-1 and GD-2 are sequences specific to T. thermosaccharolyticum GD17. JN-1 and JN-2 are sequences specific to C. thermocellum JN4. [file Image_1.TIF]

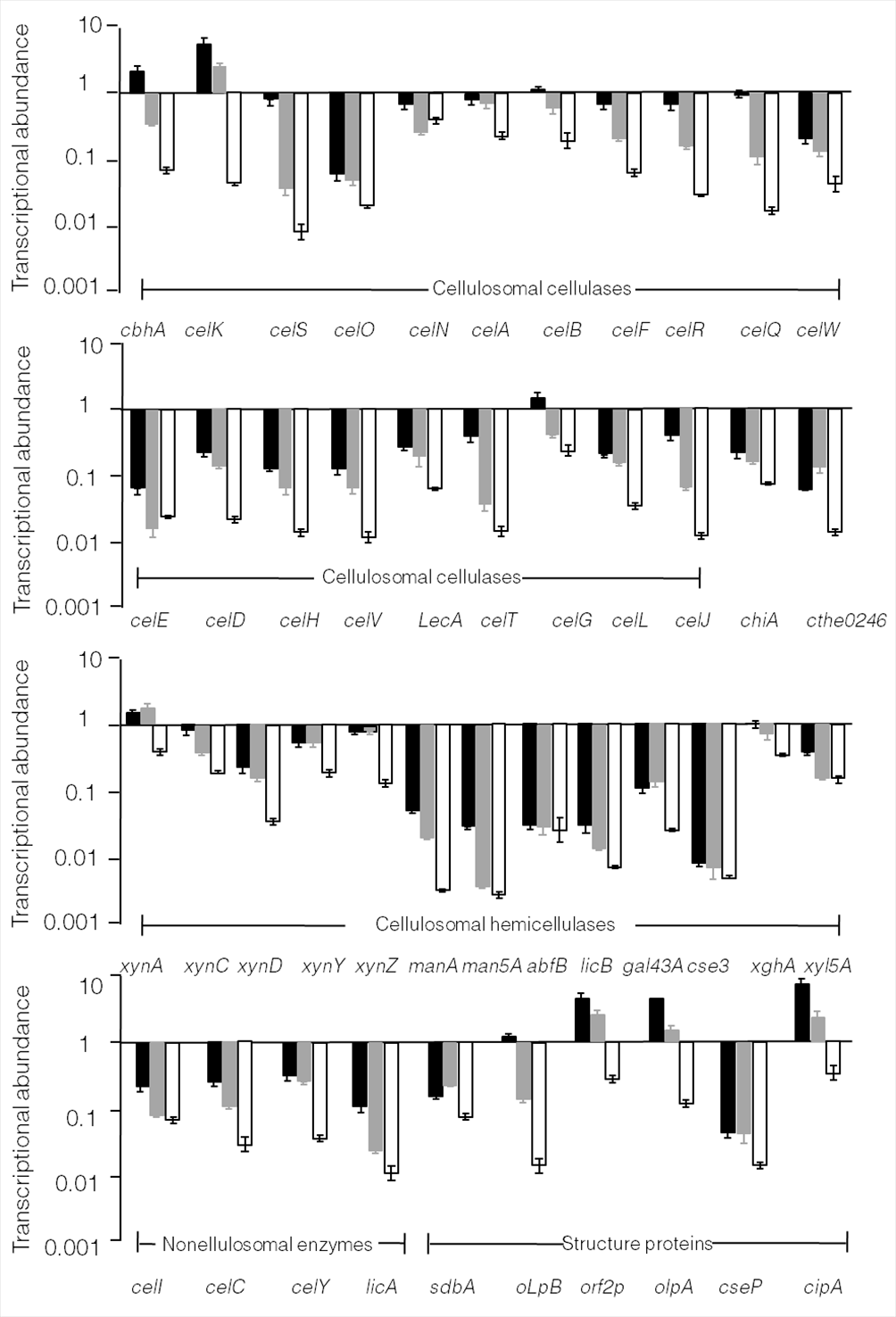

Supplement: FIGURE S2 — Comparison of expression levels of lignocellulose degradation related genes in C. thermocellum JN4 during growth on cellulose, cellobiose and glucose. Black represents cellulose, gray represents cellobiose and hollow represents glucose. Error bars are representation of standard errors calculated from nine replicates. Expression levels are normalized using recA expression as 1. [file Image_2.TIF]

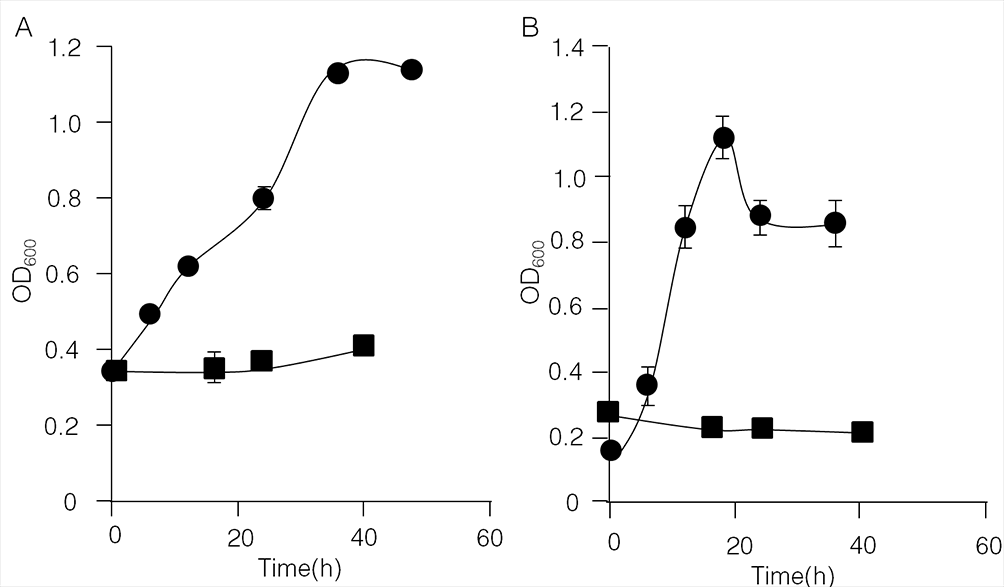

Supplement: FIGURE S3 — Growth of C. thermocellum JN4 and T. thermosaccharolyticum GD17 on dextrin and sucrose. (A) Growth on dextrin. (B) Growth on sucrose. Closed circle: T. thermosaccharolyticum GD17; closed square: C. thermocellum JN4. Error bars are representation of standard errors calculated from three replicates. [file Image_3.TIF]
