# Supplementary material for: Exploring the Relationship Between Clostridium thermocellum JN4 and Thermoanaerobacterium thermosaccharolyticum GD17
Source: Front Microbiol. 2019 Sep 10;10:2035. doi: 10.3389/fmicb.2019.02035 (PMC6746925; doi:10.3389/fmicb.2019.02035)
Supplement: TABLE S1 — Primers used in this study. [file Table_1.DOCX]

**Table S1** **Primers used in this study.**

| **Primer name** | **Targeted sequence** | **Primer sequence (5’-3’)** |
| --- | --- | --- |
| GD-1-F | GD-1 | TGGGATGTTGATTCTCTTGACTGG |
| GD-1-R | GD-1 | TGGAGTTGTTCGATAGACTGGATC |
| GD-2-F | GD-2 | ATATCAACACCATATCCAGATGTGAG |
| GD-2-R | GD-2 | ACAATGATGGCACAACAAGCGC |
| JN-1-F | JN-1 | CGCGGATCC ATGAATTTTAGCAAAAAATC |
| JN-1-R | JN-1 | CCGCTCGAGTTTATCTTTTTTTCTTGCG |
| JN-2-F | JN-2 | CGCGGATCCATGTCTTTTTTTACTATGACAAAG |
| JN-2-R | JN-2 | AAACTCGAGGCATGCTCCTGCGATAT |
| recA-F | *recA* | CAAACAGGGAAATGAGGTAATAGG |
| recA-R | *recA* | CCTGGCCGTAAATAATATCAAACTC |
| celL-F | *celL* | CACCGTCGCATTCTTTCG |
| celL-R | *celL* | TCATATCCGCCGCCTGTT |
| celN-F | *celN* | ACCCATCCCATGTCCTATGC |
| celN-R | *celN* | GCCCATTTTATTTCGTCCAG |
| celO-F | *celO* | AACAACGACGACTGGCTTCA |
| celO-R | *celO* | CGTTTGCTCCGTCAGTTTCA |
| celQ-F | *celQ* | TGGAAACAATCCGCCACA |
| celQ-R | *celQ* | CCGACCAGAGCACCGTAAA |
| celR-F | *celR* | GCTTCCCGACAACATCCGT |
| celR-R | *celR* | GGCGTCAAACCAACCTCCT |
| celS-F | *celS* | TGGAAAGTTATGGAGGATTGGA |
| celS-R | *celS* | GTTTATGAATGTTGCCCGTGTT |
| celT-F | *celT* | GATAGACTTCTGGAAACCCATACA |
| celT-R | *celT* | CAACATAGCAGCGATTCAACG |
| celV-F | *celV* | GGCTGCTGTCTGGCTTTATG |
| celV-R | *celV* | CGGCATCCCAACAGTGAA |
| celW-F | *celW* | AGCAGGATACGGTCCAAACG |
| celW-R | *celW* | CGGCAATTCGCAAAGATGA |
| lecA-F | *lecA* | TATTGAGGACGGCAAGGATG |
| lecA-R | *lecA* | TGATAAGCCCAGGCAAGTGT |
| cbhA-F | *cbhA* | CGACATCCGTGTTTGCCTTAG |
| cbhA-R | *cbhA* | CTCCATCTGTTTTGCCCTTTG |
| celA-F | *celA* | GGAGGATACAAGAGAGTACAGCGTG |
| celA-R | *celA* | GCTTCCTGCCCGTAGTTTATTG |
| celB-F | *celB* | TGACTCCAACCATCCGAACA |
| celB-R | *celB* | ACCGCCCCACCAGTTTCC |
| celD-F | *celD* | ATGATGCGGGCGACTACA |
| celD-R | *celD* | AATGCCACAGGCTCCAACT |
| celE-F | *celE* | GGGGAAATCCAAGGACAACC |
| celE-R | *celE* | CGCGTACATACCGCAGTCAAG |
| celF-F | *celF* | CGATGGCCTATGCCGTTAC |
| celF-R | *celF* | GCTCCGTCTCCCACCTGAT |
| celG-F | *celG* | TGGAAACGAAGTCTGGCTCA |
| celG-R | *celG* | GCAATGCTGGTCAGAATGGT |
| celH-F | *celH* | TCGAAGCTCCCTATGAAGGC |
| celH-R | *celH* | TCAACCACTTGCTCAACCCT |
| celJ-F | *celJ* | ATGACGACAGCGGAGACAGA |
| celJ-R | *celJ* | GGAATGCCGTAAGCCACA |
| celK-F | *celK* | TGTGATTTCGCTGTTGTTGATG |
| celK-R | *celK* | CTTCTGTCCTGGTGTAAGGTTGA |
| celP-F | *celP* | GCAGCAATCCCATCAACATG |
| celP-R | *celP* | CGCCCACCAAAGCACCTA |
| licB-F | *licB* | AAATGGAGTCGGTGGAAACG |
| licB-R | *licB* | TGCCGGGAGTAACAGGTATG |
| chiA-F | *chiA* | TTCTTACAATAGCCGCACCG |
| chiA-R | *chiA* | GAGCCAAATGATTCGCCAC |
| cse3-F | *cse3* | CCAACTCCAAGTCCGTCTCC |
| cse3-R | *cse3* | CAAAAGGCTGAAATCTGTCG |
| gal43A-F | *gal43A* | CCCGAGCCTTCACCATCA |
| gal43A-R | *gal43A* | CTGCCGTCTCCGTTCAAAT |
| manA-F | *manA* | ACATCCTTTCGGGTCAGCA |
| manA-R | *manA* | TCTTATCGCAGGCAGTTTCC |
| man5A-F | *man5A* | AAGTAGGAGCCCAGGTTTCG |
| man5A-R | *man5A* | TTGTCAGGGTCGGCGTTT |
| abfB-F | *abfB* | ATTCGCCGTTATCTGCCCTT |
| abfB-R | *abfB* | TGTTCCGTCCTCACCTTTTCC |
| Cthe0246-F | *Cthe0246* | ATTCCGACAAATGAACCACC |
| Cthe0246-R | *Cthe0246* | TACCGCCACAAGCCCTCT |
| xyl5A-F | *xyl5A* | ATGAACAGCGCATGATAGGG |
| xyl5A-R | *xyl5A* | CCGTCCGGGGACAAATAG |
| xynA-F | *xynA* | CGTCTGGGTATGAAAATGGG |
| xynA-R | *xynA* | CGGACCTGGTGGTGATGAT |
| xynC-F | *xynC* | TGCTCTGCTGACTGTCGGTAT |
| xynC-R | *xynC* | TGAATAGGAATCACCCACGAA |
| xynD-F | *xynD* | CATTTATTCTTGTTCTTTCATTGCTG |
| xynD-R | *xynD* | CACACCGTTCCAATCCTGAGT |
| xynY-F | *xynY* | GACGGATGGTGTAACTTGGG |
| xynY-R | *xynY* | ACCGTCACTCGCACTGGA |
| xynZ-F | *xynZ* | AGCGGATGTAAATAGGAGCG |
| xynZ-R | *xynZ* | CGGAGTAACAGACGGATTGG |
| xghA-F | *xghA* | GGCTGGATTGGGGAACTG |
| xghA-R | *xghA* | TTTACTTTGCCGCCTCTGTC |
| licB-F | *licB* | AAATGGAGTCGGTGGAAACG |
| licB-R | *licB* | TGCCGGGAGTAACAGGTATG |
| celC-F | *celC* | CGGTGCCTTGAGTGGTGTA |
| celC-R | *celC* | TTTTGCTGGTTCGGATCTTC |
| celI-F | *celI* | GGGACTTGCATGGCTCGAC |
| celI-R | *celI* | CATAATCCGCCTGGCTTCTT |
| celM-F | *celM* | CCCGAGGAGCAAACAGGTC |
| celM-R | *celM* | CCAAGCCAAGTCATCGCC |
| celY-F | *celY* | CAAAGTGCCGAAGGTGCC |
| celY-R | *celY* | GGATGCGGATCATAAGCCAT |
| licA-F | *licA* | ATTCAGGTAGTCCGGGCTCA |
| licA-R | *licA* | CGTTGGTCGGGTCGTCATA |
| xynX-F | *xynX* | GCTCCGCTGCTTTTTGATTC |
| xynX-R | *xynX* | CAGTTGCTCCGACAGTTCCT |
| cipA-F | *cipA* | TAGTTGTGGCTATGCTGACGAC |
| cipA-R | *cipA* | GGATCTTTTATTATGCTTCCTGGTT |
| olpA-F | *olpA* | TGAACGAAATGACCCCAAAA |
| olpA-R | *olpA* | AGAACCCGTACCGCCACC |
| olpB-F | *olpB* | AATCGGTGGAGAACATAGAGCATAC |
| olpB-R | *olpB* | CCCATGCAGCCCAGTGAGTA |
| orf2p-F | *orf2p* | TTTACTTTGCCGCCTCTGTC |
| orf2p-R | *orf2p* | TGCATCCCAGTCAAACAGC |
| sdbA-F | *sdbA* | GGATGTGCTGAGTTTGGGTGAC |
| sdbA-R | *sdbA* | GCGTGGATGACGGAGTTGC |
| cseP-F | *cseP* | TGGGAAGCGAGGGAGAAA |
| cseP-R | *cseP* | TGGGAAGGGTGTCGGAGT |
